# Sleep Inducing for EEG Recording in Children: A Comparison between Oral Midazolam and Chloral Hydrate

**Published:** 2013

**Authors:** Mahmoud Reza ASHRAFI, Reza AZIZI MALAMIRI, Gholam Reza ZAMANI, Mahmoud Mohammadi, Firozeh HOSSEINI

**Affiliations:** 1 Professor of Pediatric Neurology, Pediatrics Centre of Excellence, Department of Pediatric Neurology, Children’s Medical Centre, Tehran University of Medical Sciences, Tehran, Iran; 2 Assistant Professor of Pediatric Neurology, Department of Pediatric Neurology, Golestan Medical, Educational, and Research Centre, Ahwaz Jundishapur University of Medical Sciences, Ahwaz, Iran; 3 3.Associate Professor of Pediatric Neurology, Pediatrics Centre of Excellence, Department of Pediatric Neurology, Children’s Medical Centre, Tehran University of Medical Sciences, Tehran, Iran; 4 Assistant Professor of Pediatric Neurology, Department of Pediatric Neurology, Besat Hospital, Hamadan University of Medical Sciences, Hamadan, Iran

**Keywords:** Chloral hydrate, Oral midazolam, Sleep, Sedation, Electroencephalography, Children

## Abstract

**Objective:**

Electroencephalography (EEG) recording is a long duration procedure that needs patient’s cooperation for device setup and performing the procedure. Many children lose their cooperation during this procedure.

Therefore, sedation and sleep are frequently induced using a few agents as pre procedure medication in children before EEG recording. We aimed to compare the sedative effects of oral midazolam versus chloral hydrate before the procedure along with their impacts on EEG recording in children.

**Materials & Methods:**

A randomized trial was carried out to compare the sedative effects of oral midazolam versus chloral hydrate and their impacts on EEG recording in children. A total of 198 children (100 in the midazolam group and 98 in the chloral hydrate group) were enrolled in the study and randomly allocated to receive either oral moidazolam or chloral hydrate.

**Results:**

Oral midazolam had superiority neither in sleep onset latency nor in sleep duration when compared to chloral hydrate. Moreover, the yield of epileptiform discharges in the chloral hydrate group was more than the midazolam group.

**Conclusion:**

The results of this study showed that both chloral hydrate 5% (one ml/kg) and oral midazolam (0.5 mg/kg) could be administered as a pre medication agent for EEG recording in children. However, oral midazolam at this dose had no advantage compared with chloral hydrate.

## Introduction

Performing procedures in children are difficult due to no cooperation and fear of procedures. Therefore, sedation and sleep are frequently induced using some agents as pre procedure medication in children. EEG recording is a long duration procedure and needs patient cooperation for device setup and performing the procedure.

Because of long duration of the EEG recording, many children lose their cooperation and begin to move or cry during the procedure and due to lack of cooperation, EEGs are recorded poorly and are difficult to interpret by neurophysiologists (1,2).

An ideal pre medication for inducing sedation and sleep in children who are candidates for EEG recording should rapidly induce sleep and because of long duration of EEG recording, this agent should have a long acting hypnotic effect. Furthermore, such agent should also have no or minimal effect on the EEG background and paroxysmal transients in the EEG. Because of ease of use, oral medications are far superior to parenteral agents (1-3). 

Chloral hydrate is a sedative and hypnotic medication that has been used since 1869. This medication has been used for a long time in our center to induce sleep in children who are referred for EEG recording and have no cooperation for procedure performing. Chloral hydrate has two active metabolite, Trichloroethanol (TCE) and Trichloroacetic acid (TCA). By an unknown mechanism, TCE exerts sedative and hypnotic effects of chloral hydrate on the CNS. Chloral hydrate has long been used as pre-medication for EEG recording, however, this medication has many known adverse effects such as; nausea, vomiting, agitation, ataxia, prolonged sedation, delayed apnea events, gastric irritation, potential carcinogenicity, and genotoxicity even as a single low dose (1,3,4).

Midazolam is a sedative, hypnotic, and anticonvulsant agent, which has been used as a pre medication agent in many procedures in children (5-10). Recently, oral solution of this medication has been available in our country (Iran), and anecdotal reports have proposed its efficacy as a pre medication in intubation and endoscopy. However, to the best of our knowledge, no study has yet been performed to show the superiority of oral midazolam as a pre medication for EEG recording in children. Therefore, we carried out a randomized trial to compare the sedative effects of oral midazolam versus chloral hydrate and their impacts on EEG recording in children.

## Materials & Methods

**Study location, sample, and design**

Our study was conducted between May 2010 and May 2011 in a major University Paediatric Hospital in Tehran, Iran. We enrolled children aged between one month and ten years who were referred for EEG recording and were uncooperative with the device setup or were referred to our center for sleep EEG recording. We excluded children if they had hypersensitivity to midazolam or chloral hydrate, hepatic disease, peptic ulcer, respiratory disease or received medications that had dangerous interactions with midazolam or chloral hydrate. 198 Consecutive patients were enrolled and randomly assigned to receive either oral moidazolam (midazolam group, n=100) or chloral hydrate (chloral group, n=98). Parents were asked to awaken their children at 6.00 am and not to let them fall asleep until the time of EEG recording. 

Midazolam was given at a dose of 0.5 mg/kg and Chloral hydrate 5% was given at a dose of one ml/kg of body weight orally, one hour before EEG recording. A trained staff filled out a questionnaire for each patient to collect the following data: neurological diagnosis, sleep onset latency, sleep duration, drowsiness time, and adverse events that occurred in the first 24 h after EEG recording. We used an analogue EEG machine (Nihon Kohden) in our center for EEG recording, and EEG was recorded using 21 scalp electrodes based on the standard international 10 20 system. A trained and skilled child neurophysiologist interpreted the recorded EEGs.

**Ethics**

The study protocol was approved by the Ethics Committee of Tehran University of Medical Sciences, and a written informed consent approved by the Institutional Review Board was obtained from all parents prior to enrolment in the study. The trial was registered with the Ethics Committee of Tehran University of Medical Sciences prior to any patient enrolment.

**Statistical analysis**

Based on previous data and using two-tailed tests, the sample size was calculated keeping Type I error (α)=0.05 and Type II error (β)=0.2. The necessary sample size was calculated to be at least 100 patients in each group in order to detect any difference between two groups, with the power of the test set at 80%. Data were recorded in forms previously explained to trained staff. Recorded data were assessed for normal distribution using the Shapiro Wilk test. Statistical analysis was performed using independent samples t-test and Mann Whitney Rank Sum test for continuous data. Categorical variables were analyzed using Fisher exact and 2 Tests. A value of p<0.05 was considered significant. A biostatistician who was blinded to the study groups performed statistical analysis.

## Results

The midazolam group consisted of 47 boys and 53 girls, with the median age of 4 years and a range from 2 month to 9 years. The chloral hydrate group consisted of 48 boys and 50 girls with the median age of 4 years with a range of 3 months to 10 years. In the midazolam group, 80 children were referred for EEG recording due to seizure disorders, 15 because of developmental delay, and five due to autistic feature. In the chloral hydrate group, 79 children were referred for EEG recording due to seizure disorders, 13 for developmental delay, and six due to autism. No significant difference was seen between two groups concerning referral etiologies ( = 0.22, df=2; p=0.896).

Sleep onset latency was significantly shorter in the chloral hydrate group than midazolam group [between 45 and 98 min (a median of 58 min) in the midazolam group and between 20 and 95 min (a median of 32 min) in the chloral hydrate group [Mann-Whitney U Statistic=1940.000, T=6791.000, n (small)=98 n (big)=100; (p<0.001)]. Sleep duration was significantly longer in the chloral hydrate group compared to midazolam group [between 12 and 38 min (a median of 25.5 min) in the midazolam group and between 56 and 98 min (a median of 66.5 min) in the chloral hydrate group [Mann-Whitney U Statistic=0.000, T=14651.000, n(small)=98 n(big)=100; (p<0.001)]. Drowsiness time was significantly shorter in the midazolam group than chloral hydrate [between 2 and 9 min (a median of 6 min) in the midazolam group and between 21 and 36 min (a median of 32 min) in the chloral hydrate group [Mann-Whitney U Statistic=0.000, T=14651.000 n(small)=98 n(big)=100; (p<0.001)] (Table 1). 

**Table 1:** Sleep Characteristics Between Groups of Chloral Hydrate and Oral Midazolam

**Sleep onset latency** a		**p-value** b	**Sleep durationa**		**p-value** b	**Drowsiness time** a		**p-value** b
Chloral hydrate range (median)	Midazolam range (median)		Chloral hydrate range (median)	Midazolam range (median)		Chloral hydrate range (median)	Midazolam range (median)	
20-95 (32)	45-98 (58)	<0.001	56-98 (66.5)	12-38 (25.5)	<0.001	21-36 (32)	2-9 (6)	<0.001

a : Values are expressed in minutes

b : P-value using Mann-Whitney U test

Abnormal epileptiform discharges were significantly more reported in children who sedated by chloral hydrate than midazolam [85 children in chloral hydrae group (87%) and 45 children in midazolam group (45%),  ^2^=36.404, df =1; p<0.001]. The most frequent drug effect on EEG was generalized fast beta activity followed by slow delta activity in temporal regions.

These drug’s effects on EEG were significantly more reported in children who received midazolam as pre medication agent [40 children in chloral hydrae group (41%) and 70 children in midazolam group (70%),  ^2^=15.911, df=1; p< 0.001] No significant adverse reactions were seen in both groups but two children in the midazolam group developed short duration irritability and were improved without using other medication.

## Discussion

EEG recording usually needs induced sedation and sleep in children who uncooperative with the device setup and EEG recording. The ideal pre medication for EEG recording in children should rapidly induce sleep and because of long duration of the EEG recording should have a long acting hypnotic effect. Furthermore, this pre medication agent should also have no or minimal effect on EEG background and paroxysmal transients in EEG (1-3). 

Results of the present study indicated that both chloral hydrate and oral midazolam showed the characteristics of a suitable pre medication agent for EEG recording in children.

However, oral midazolam had superiority neither in sleep onset latency nor in sleep duration when compared to chloral hydrate. Moreover, the yield of epileptiform discharges in the chloral hydrate group was more than the midazolam group. On the other hand, results of this study showed that drowsiness time from midazolam was shorter than chloral hydrate, although this effect has no place in EEG recording and adds some difficulty for parents to handle their children after EEG recording. Therefore, based on the results of this study, chloral hydrate is still a more suitable choice as a pre medication agent for EEG recording in children.

The results of this study are comparable with those of previous studies in children. In a similar study, melatonin versus chloral hydrate was assessed as pre medication agents for EEG recording in children. In the mentioned study 384 children aged 1 to 72 months were enrolled to evaluate sleep onset latency, sleep duration, and drowsiness time along with the yield of epileptiform discharges (1). Results of that study showed that both melatonin and chloral hydrate are valuable pre medication agents in children. Other studies with different methodologies in children showed the beneficial effects of oral midazolam such as decreased anxiety and acceptable sedation before upper gastrointestinal endoscopy or intubation (2,3,5-14).

To the best of our knowledge, our study is the first study that compares the effects of oral midazolam with those of chloral hydrate. However, the results of our study should be interpreted in the face of certain limits. We administered oral midazolam at a dose of 0.5 mg/kg for each children and this dose may explain longer sleep latencies and shorter sleep durations in the children of midazolam group. We think higher doses could improve sleep latency and sleep duration.

In conclusion, results of our study indicate that both chloral hydrate 5% (one ml/kg) and oral midazolam (0.5 mg/kg) can be administered as a pre medication agent for EEG recording in children. However, oral midazolam at this dose had no superiority to chloral hydrate. 
